# Genetic Association Study of Acetylcholinesterase (*ACHE*) and Butyrylcholinesterase (*BCHE*) Variants in Sudden Infant Death Syndrome (SIDS)

**DOI:** 10.3390/genes15121656

**Published:** 2024-12-23

**Authors:** Dong Qu, Peter Schürmann, Thomas Rothämel, Thilo Dörk, Michael Klintschar

**Affiliations:** 1 Institute of Legal Medicine, Hannover Medical School, Carl-Neuberg-Str. 1, 30625 Hannover, Germany; qudong_fimmu@hotmail.com (D.Q.); rothaemel.thomas@mh-hannover.de (T.R.); 2 Department of Forensic Medicine, School of Basic Medical Sciences, Nanjing Medical University, Longmian Avenue 101, Nanjing 211166, China; 3 Gynaecology Research Unit, Hannover Medical School, Carl-Neuberg-Str. 1, 30625 Hannover, Germany; schuermann.peter@mh-hannover.de (P.S.); doerk.thilo@mh-hannover.de (T.D.)

**Keywords:** sudden infant death syndrome (SIDS), genetic association, polymorphisms, *ACHE*, *BCHE*

## Abstract

Background: Sudden infant death syndrome (SIDS) is the leading cause of death among infants aged between one month and one year. Altered enzyme activities or expression of acetylcholinesterase (AChE) and butyrylcholinesterase (BChE) have been observed in SIDS patients that might lead to disturbed autonomic function and, together with other risk factors, might trigger SIDS. To explore the contribution of AChE and BChE from a genomic viewpoint, we sought to investigate the association between SIDS and selected single nucleotide polymorphisms (SNPs) in the *ACHE* and *BCHE* genes. Methods: In this case-control study, 13 potentially regulatory SNPs were selected from *ACHE* and *BCHE* and were genotyped in 201 SIDS cases and 338 controls. The association of SIDS with the 11 successfully genotyped candidate variants was examined using statistical analyses of overall or stratified cases and haplotype analyses. Results: No significant overall associations were observed between SIDS and *ACHE* and *BCHE* variants in allele, genotype, and haplotype analyses. In subgroup analyses, eight variants were found to be nominally associated with SIDS, though these associations did not remain statistically significant after correction for multiple comparisons. One haplotype (T-C-G-C-C in rs3495-rs1803274-rs1355538-rs2048493-rs1126680) of *BCHE* was associated with the female SIDS subgroup (57.3% in controls vs. 46.3% in female SIDS cases, *p* = 0.010). Conclusions: The selected variants in *ACHE* and *BCHE* were not overall associated with SIDS in this study, and thus cannot generally explain the previously reported dysregulation of enzyme activities in SIDS. However, some evidence of association in subgroups and a possible contribution of variants other than those tested here would need to be explored in larger studies.

## 1. Introduction

Sudden infant death syndrome (SIDS) is the primary cause of mortality among infants aged one month to one year in developed countries [1]. According to the triple-risk model, SIDS is associated with the convergence of three factors, namely, a crucial developmental stage, an infant susceptible to harm, and external factors [2]. Although this model, which is not generally embraced [3], can explain several phenomena associated with SIDS, the underlying causes of SIDS have yet to be better defined. As a matter of fact, widely varying etiological factors have been proposed, among those, e.g., impaired development of the immune system [4], cardiac arrhythmia syndromes [5], or an abnormal stress response [6]. Among the most prominent neuropathological mechanisms discussed is the serotonergic system [7], for which genetic abnormalities are reported [8]. However, the autonomic nervous system is complex and many neurotransmitters are involved.

Acetylcholine (ACh) plays a role in various regions of the central nervous system (CNS) [9]. Within the CNS, ACh modulates the communication between various neurons in the brain regions that regulate motivation, arousal, and attention [10,11,12]. The synthesis of ACh primarily occurs at the presynaptic membrane, catalyzed by choline acetyltransferase through the synthesis of choline and acetyl coenzyme A, which are subsequently released into the synaptic cleft and act on muscarinic (M) and nicotinic (N) receptors located on the postsynaptic membrane, resulting in physiological effects [13,14]. Subsequently, ACh dissociates from its receptors and is inactivated by hydrolysis through cholinesterase (ChE) in the synaptic cleft, resulting in the formation of choline and acetic acid. A fraction of the choline is subsequently reabsorbed and reused by the presynaptic membrane choline transporter [15].

Humans possess two choline esterases, acetylcholinesterase (AChE) and butyrylcholinesterase (BChE). AChE and BChE play a vital role in maintaining the homeostasis of the cholinergic system, whose dysregulation is thought to be involved in the pathogenesis of SIDS [16,17,18,19,20,21]. Several studies have investigated the enzyme activity or expression of AChE in multiple tissues of SIDS; however, diverse results were unveiled [17,18,19,20,21]. Livolsi et al. reported an increased enzyme activity of AChE in the red blood cells (RBCs) of SIDS cases [21]. On the other hand, Dick and Ford found slightly decreased activities of AChE in the RBCs of SIDS cases, although these differences were not statistically significant [18]. Moreover, levels or activities of AChE have been determined in the CNS of SIDS cases; however, diverse findings were observed [17,19,20]. All these above-mentioned investigations were performed posthumously. However, the interest in the cholinergic transmitter system was recently renewed when Harrington et al. reported a decreased activity of BChE in postnatally taken blood-dried spots [16]. Should these results be confirmed in future experiments, this would be the first marker during life to inform on the risk of developing SIDS; however, the mechanisms that lead to the reported diminished activity of BChE remain to be clarified [16]. Moreover, the authors could not obtain results for AChE due to storage conditions [16]; however, from a theoretical point of view, both cholinesterases could contribute to SIDS.

A genetic component is suspected in SIDS [22], and cholin esterase activity has been linked with genomic variants of *ACHE* and *BCHE* in multiple diseases [23,24,25]. Nevertheless, up to now, no genetic study on an association of SNPs in *ACHE* and *BCHE* genes with SIDS has been performed. Thus, we hypothesized that regulatory variants in *ACHE* and *BCHE* genes might participate in the etiology of SIDS via altering enzyme expression or in the activity of ChEs and, subsequently, cause cholinergic dysfunctions. To this end, four known candidate variants from *ACHE* and nine variants from *BCHE* were selected and genotyped in 201 SIDS cases and 338 controls to elucidate potential genetic links between SIDS and the imbalance of the cholinergic system.

## 2. Materials and Methods

### 2.1. Study Subjects

The SIDS group (N = 201) was recruited from the Institute of Legal Medicine at Hannover Medical School, Germany, as previously described [26]. Of the SIDS cases, 60.5% were male and 39.5% female. All were of Caucasoid origin. The control group (N = 338) included 175 males and 163 females, comprising 33 children and 305 healthy adults. The 33 children included in the control group were in their first year of life but died from explicit causes other than SIDS, such as trauma, infections, and congenital heart defects. This study has been approved by the ethics commission of Hannover Medical School (approval code: 1211-2011, approval date: 14 October 2011).

### 2.2. Selection of SNPs and Genotyping

**Figure 1:**
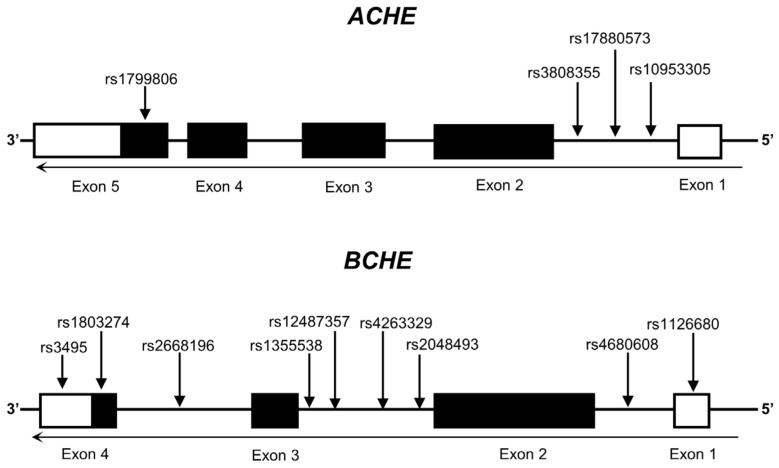
Genomic structure of human *ACHE* and *BCHE*, highlighting the locations of selected SNPs. Exons are represented by boxes, untranslated regions are depicted in white, and translated regions are delineated in black.

Candidate SNPs for *ACHE* and *BCHE* were selected on the basis of the available literature describing putative relevance to specific diseases or possible effects on gene expression. Selection criteria included (1) minor allele frequency (MAF) equal to or greater than 0.05 in European populations, (2) the SNPs were not in high linkage disequilibrium (LD) (R^2^ < 0.8), and (3) the SNPs were documented in previously published studies or in the GTEx portal (https://www.gtexportal.org/home/, accessed on 10 October 2024), indicating their potential effect on the expression of the gene of interest. In total, 13 candidate regulatory variants (four in the *ACHE* gene and nine in the *BCHE* gene) were identified and included in the analysis. Detailed information about these 13 SNPs is shown in Figure 1 and Table 1.

Blood (whole blood) and thymus samples from the decedent were collected during autopsies and then stored at −20 °C. Buccal swabs were used to collect saliva from the living and then were air-dried and stored at room temperature. Only autopsies from bodies without marked postmortem changes were included. DNA was isolated from blood, saliva, or thymus samples following the manufacturer’s instructions for the QIAamp DNA Mini Kit (Cat#: 51306, Qiagen, Hilden, Germany). All DNA samples were stored at −20 °C for long-term preservation. Genotyping was performed using the Fluidigm Biomark EP1 platform (Fluidigm, South San Francisco, CA, USA) with 192.24 Dynamic Array™ IFC for SNP Genotyping (Cat#: BMK-M-192.24GT, Fluidigm, South San Francisco, CA, USA), as described in our previous studies [27]. The raw data was transformed and analyzed using the Fluidigm SNP Genotyping Analysis Software (Version 4.5.1). The probes and related primers used in the study are listed in Supplementary Table S1.

### 2.3. Statistical Analyses

The assessment of Hardy–Weinberg equilibrium (HWE) in the control group was conducted using an online HWE calculator, available at https://wpcalc.com/en/equilibrium-hardy-weinberg/ accessed on 10 October 2024. The evaluation of the association between SNPs and SIDS was carried out using a 2 × 2 Chi-square (χ^2^) test, applying both dominant and recessive models. Additionally, a linear-by-linear model of the χ^2^ test was applied under the additive model. Subsequently, odds ratios (ORs), 95% confidence intervals (CIs), and corresponding *p*-values were computed.

In the stratified analysis, the participants were categorized into four groups, delineated by their respective risk factors associated with SIDS. These groups were defined by (1) sex (118 males, 83 females), (2) age group (0–4 months: 103 subjects, 2–4 months: 75 subjects, 4–8 months: 48 subjects, 8–12 months: 7 subjects), (3) time of death season (spring: 44 subjects, summer: 31 subjects, spring + summer: 75 subjects, autumn: 43 subjects, winter: 42 subjects, autumn + winter: 85 subjects), and (4) position the baby is found in after death (prone position: 45 subjects, other positions: 15 subjects).

**Figure 2:**
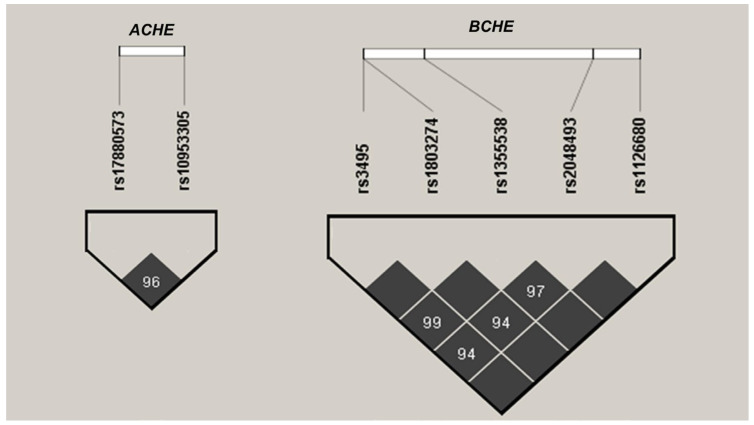
Linkage disequilibrium (LD) patterns in the *ACHE* and *BCHE* genes in a cohort of 539 subjects. Black squares denote regions of strong LD. The values within the cells indicate pairwise degrees of LD, expressed as D’ × 100.

For haplotype analysis, SNPs in linkage with each other among the European population (with R^2^  <  0.8 and D′  >  0.75) were included. Haplotype patterns among the study subjects were analyzed using Haploview 4.2 (Broad Institute, Cambridge, MA, USA). The identified haplotype blocks of *ACHE* and *BCHE* of this study are displayed in Figure 2.

In all statistical analyses, nominal statistical significance was attributed to a two-sided *p*-value < 0.05. To address the issue of multiple comparisons, the Bonferroni correction was applied. Significance after multiple testing was established at *p* < 0.0001 [α = 0.05/(15 strata × 11 assays × 3 genetic effects)]. The statistical computations were executed utilizing SPSS 24.0 software (SPSS Inc., Chicago, IL, USA).

### 2.4. Expression Quantitative Trait Loci (eQTL) Analysis for Enzyme Expression

eQTL are genetic loci that explain variation in expression levels of mRNAs by integrating genotypic data with the mRNA expression data from transcriptomic sequencing studies from multiple human tissues. Specific information about the eQTL project is described elsewhere [28]. To assess if the focused SNP locus might have an impact on the gene expression and where it is located, we accessed the data from the GTEx-eQTL browser (https://gtexportal.org/home/, accessed on 10 October 2024).

## 3. Results

We genotyped 13 variants at *ACHE* and *BCHE* in up to 201 SIDS cases and 338 controls. After QC, we excluded one SNP (rs3808355 in *ACHE*) with a low call rate (<95%). Among the remaining 12 SNPs, the call rate for the whole assays and the concordance rate among 31 technical duplicates were 99.9% and 96.8%, respectively. One SNP (rs1799806 in *ACHE*) deviating from HWE was eliminated from the subsequent analysis.

The overall analysis (Table 2) showed no significant associations between the remaining 11 SNPs and SIDS. In stratified analyses (Table 3), eight out of eleven SNPs were found to be associated with SIDS at the subgroup levels. Six variants (rs4680608, rs1355538, rs3495, rs1803274, and rs2048493), all in *BCHE*, were found to be associated with SIDS in the female subgroup. In age-stratified analyses, rs3495 and rs4263327, located in the *BCHE* gene, were linked to SIDS cases who died at the age of 2~4 months. Three SNPs (rs2048493, rs4680608, rs12487357) from *BCHE* were unveiled to associate with SIDS in autumn, and one SNP in *ACHE* (rs10953307) appeared associated with SIDS occurring in summer.

To assess the influence of the above-mentioned eight SIDS-associated SNPs (one in *ACHE* and seven in *BCHE*) on their enzyme expressions, eQTL analysis results from the liver, whole blood, and brain subregions were obtained from the GTEx database (Supplementary Table S2). The minor allele of the *ACHE* SNP (rs10953305) was associated with higher *ACHE* mRNA expression in the brain, lower mRNA expression of *ACHE* in the liver, and no changes in whole blood. Furthermore, the female risk alleles of rs1355538, rs2048493, and rs4680608 were associated with increased mRNA expression of *BCHE* in the liver or/and specific brain subgroups. None of the subgroup risk-related SNPs in *BCHE* were linked to altered mRNA expression of *BCHE* in whole blood.

As a caveat, all nominally significant results were not retained after the Bonferroni correction for multiple comparisons (*p* < 0.0001). To evaluate the combined effect of SNPs on SIDS, haplotype-based association analyses were conducted. No overall significant results were obtained; however, one haplotype (T-C-G-C-C in rs3495-rs1803274-rs1355538-rs2048493-rs1126680) of *BCHE* was associated with the female SIDS subgroup (57.3% in controls vs. 46.3% in female SIDS cases, *p* = 0.010) (Table 4 and Table 5).

## 4. Discussion

AChE and BChE, as cholinesterase (ChE), inactivate acetylcholine through hydrolysis to maintain the homeostasis of the cholinergic system, whose disturbance would cause impairment in motivation, arousal, and attention [10,11,12], with arousal being discussed to be a factor in the pathogenesis of SIDS [29]. Previously altered expression or activity of AChE and BChE in the tissues of SIDS cases were reported [15,17,18] and, most importantly, newborns destined to die from SIDS might have a diminished activity of BChE [15]. However, the cause for these abnormalities is still not clear. In a context different from SIDS, some studies have unveiled associations between enzyme expressions/activities and certain regulatory SNP loci in *ACHE* and *BCHE* [22,23,24,25]. This prompted us to investigate for the first time whether such SNPs in *ACHE* and *BCHE* are associated with SIDS and whether they could further explain and eventually predict the AChE and BChE alterations reported by Harrington et al. and other groups from a viewpoint of genetics.

To that end, 11 SNPs (nine from *BCHE* and two from *ACHE*) were successfully genotyped and analyzed in 338 controls and 201 SIDS cases. All of these SNPs had been previously associated with specific diseases and altered enzyme expressions or activities in related articles or the GTEx database, indicating that they might be functionally relevant to the cholinergic system. Nevertheless, no statistically significant association with SIDS was detected in our overall analysis. However, in the stratified analysis, eight (seven from *BCHE* and one from *ACHE*) out of eleven SNPs were nominally associated with at least one subgroup, including SIDS among female babies for six *BCHE* variants, although statistical significance vanished after Bonferroni correction for multiple comparisons. Moreover, one haplotype was found to be relevant to the female SIDS subgroup, which suggests a combined impact of *BCHE* SNPs on the etiology of SIDS. Nevertheless, the exact functional consequence of this identified haplotype is unknown so far. The findings suggest that the selected SNPs from the *ACHE* and *BCHE* genes are of limited relevance in SIDS while the role of *BCHE* variants in female SIDS patients may warrant further investigation. Although previous studies have found that certain genetic polymorphisms are predominantly associated with females, most of the relevant studies have focused mainly on explaining the excess of males. One might thus hypothesize that male and female SIDS differs to some extent: SIDS is believed to include numerous different etiologies. The quantitative distribution of these etiologies might well differ between males and females, and genetic associations specifically with female SIDS cases might be warranted.

BChE as a biological enzyme has its activity primarily determined by both the quantity and structure of the enzyme. The gross quantity but not final activity of BChE can be, to some extent, represented by the level of its mRNA that could represent the concentration of BChE [30]. Considering this, the impact of the SIDS subgroup-related risk SNPs screened in this study on expression levels of BChE was evaluated by in silico analyses. As it has been demonstrated that BChE was primarily generated in the liver and circulated through the bloodstream, the possible role of *BCHE* SNPs in gene expression was checked in the liver and whole blood samples using the GTEx portal. Moreover, it has been postulated that the dysregulation of BChE in the CNS might be involved in triggering SIDS; therefore, the effects of *BCHE* SNPs on mRNA levels were also assessed in multiple brain subregions. The risk alleles of all the seven *BCHE* SNPs (rs4680608, rs1355538, rs3495, rs1803274, rs2048493, rs4263329, and rs12487357) associated with SIDS subgroups showed no regulatory potentials on mRNA expression of *BCHE* in whole blood based on the public data from the GTEx portal. Regarding the findings in livers and brain subregions, two out of seven *BCHE* SNPs (rs1355538 and rs2048493) demonstrated an association with increased mRNA levels of *BCHE*. Thus, the results of the in silico analysis using the GTEx database do not appear to provide strong support for the hypothesis that the SNPs identified in certain SIDS subgroups of this study are responsible for the observed decreased activity of BChE in the blood of SIDS cases via decreasing the expression of BChE.

However, risk alleles for rs3495, rs1803274, and rs4263329 of *BCHE* have been reported to be associated with a low activity of plasma BChE in other diseases [22,23,24,25]. Meanwhile, in the stratified analysis of this study, all SIDS-associated risk alleles or genotypes of these three *BCHE* SNPs had a higher proportion in SIDS cases than in controls, indicating a possibly decreased activity of BChE triggered by these three SNPs might exist in some subsets of SIDS. This would be partially in line with the previously reported significant results, showing a decreased BChE activity in SIDS cases [16]. It must, however, be borne in mind, that the rather common polymorphisms studied herein only determine a small part of the variation in enzyme activity observed in the population. Even more so, it could not easily explain changes during the lifetime of an individual, as the genome (as opposed to, e.g., DNA methylation) does not change.

Therefore, from a genetic point of view, our results cannot fully explain the previously reported alterations in the enzymatic activity or expression of AChE or BChE in SIDS cases. It is noteworthy that only a limited number of polymorphisms for each gene were included in our study and two variants technically failed; therefore, the participation of other relevant SNPs in SIDS cannot be excluded.

As mentioned above, according to the triple-risk hypothesis [3], SIDS is triggered only when there is an overlapping between a critical development, a vulnerable infant (e.g., genetic impacts), and the influence of external risk factors. Besides genetic impacts as an important basis for the pathogenesis of SIDS, external factors also play a vital role in triggering SIDS. It must thus be borne in mind that altered expressions or activities of BChE could be impacted not only by genetic facets but also by diseases and unhealthy conditions [31], such as CNS disorders [32], liver disease [33], inflammation [34], and smoking [35]. Recently published papers regarding SIDS have pointed out that infection might play a vital role in the etiology of SIDS from multiple facets [36,37,38,39]. As mentioned above, inflammation may lead to changes in BChE expression or activity in other diseases. This way, infection and inflammation are among the risk factors for SIDS and may also contribute to dysregulated BChE levels in cases of SIDS. Furthermore, it has been suggested that there might be a possible interaction among cholinergic disorders, serotonergic dysfunction, and maternal smoking in the etiology of SIDS [40]. Notably, it is well known that individuals with a BChE deficiency caused by genetic mutations exhibit an absence of symptoms, with the exception of an increased susceptibility to the muscle relaxants suxamethonium and mivacurium [41,42]. In other words, complete BChE deficiency does not result in disease unless both the above-mentioned medications and improper treatments are administered. Hence, the previously documented reduction in BChE activity alone would not be able to explain the occurrence of SIDS. However, together with additional underlying risk factors (e.g., maternal smoking) or etiologies (e.g., serotonergic dysfunction) as suggested by the triple risk hypothesis, it could easily explain SIDS. Nevertheless, these considerations do not impede the potential of BChE as a biomarker for SIDS.

## 5. Conclusions

In summary, no overall associations were found between SIDS and the selected SNPs from *ACHE* and *BCHE*, and, thus, our results cannot generally explain the previously reported alterations in the enzyme expression or activity of AChE and BChE in SIDS. Though we found evidence that some *BCHE* variants may be associated with female SIDS or seasonal effects, these were not maintained statistically. In the future larger studies, investigating associations between more genetic variants from *ACHE* and *BCHE* might shed a brighter light on this question. Even more so, it might be worthwhile to further explore the potential impact of external risk factors (e.g., maternal smoking) or underlying etiologies (e.g., serotonergic dysfunction) of SIDS on the expression or activity of AChE and BChE.

## Figures and Tables

**Table 1:** General information of selected SNPs from *ACHE* and *BCHE*.

SNP	Alleles	Genome Position (GRCh38)	Gene: Consequence	Reported Association with Other Diseases?	Altered Gene Expression in eQTL
rs3495	T > C	chr3:165773193	*BCHE*: Non-Coding Transcript Variant	Yes	*BCHE*
rs1126680	C > T	chr3:165837337	*BCHE*: Non-Coding Transcript Variant	Yes	*BCHE*
rs1803274	C > T	chr3:165773492	*BCHE*: Missense Variant	Yes	*BCHE*
rs1355538	G > A	chr3:165787389	*BCHE*: Intron Variant	Yes	*BCHE*
rs2048493	C > G	chr3:165826514	*BCHE*: Intron Variant	Yes	*BCHE*
rs2668196	T > A	chr3:165784921	*BCHE*: Intron Variant	Yes	*BCHE*
rs4263329	A > G	chr3:165821822	*BCHE*: Intron Variant	Yes	*BCHE*
rs4680608	T > C	chr3:165835858	*BCHE*: Intron Variant	Yes	*BCHE*
rs12487357	G > C	chr3:165804028	*BCHE*: Intron Variant	Yes	None
rs1799806	G > C	chr7:100891037	*ACHE*: Missense Variant	Yes	*ACHE*
rs3808355	C > T	chr7:100894677	*ACHE*: Intron Variant	No	*ACHE*
rs17880573	C > T	chr7:100894791	*ACHE*: Intron Variant	No	*ACHE*
rs10953305	G > A	chr7:100894947	*ACHE*: Intron Variant	No	*ACHE*

Note: Major allele > minor allele. eQTL: Expression quantitative trait loci.

**Table 2:** Overall analysis of the association between the SNPs from *ACHE* and *BCHE* genes and SIDS.

Gene	SNP	Alleles	Genotype Distribution (XX:XY:YY)		*p* Value		
			SIDS	Control	Additive	Dominant	Recessive
*BCHE*	rs3495	T > C	109:71:21	188:123:27	0.625	0.753	0.351
	rs1126680	C > T	149:45:07	239:91:05	0.938	0.793	0.900
	rs1803274	C > T	133:60:08	231:92:15	0.795	0.635	0.799
	rs1355538	G > A	73:83:44	114:162:62	0.321	0.514	0.314
	rs2048493	C > G	88:84:29	153:142:43	0.844	0.737	0.602
	rs2668196	T > A	135:61:5	218:107:13	0.637	0.529	0.466
	rs4263329	A > G	168:29:04	284:51:02	0.324	0.832	0.203
	rs4680608	T > C	136:51:14	233:93:12	0.204	0.774	0.095
	rs12487357	G > C	176:23:02	284:53:01	0.235	0.314	0.559
*ACHE*	rs1799806	G > C	Deviation from HWE		-	-	-
	rs3808355	C > T	Low call rates		-	-	-
	rs17880573	C > T	149:45:07	239:91:05	0.181	0.549	0.132
	rs10953305	G > A	46:102:51	91:157:89	0.513	0.357	0.919

Note: Major allele > minor allele. X and Y, respectively, represent major and minor alleles for corresponding SNPs. HWE: Hardy–Weinberg Equilibrium. Nominal *p* value less than 0.05 was regarded as statistically significance.

**Table 3:** Selected SNPs associated with subgroups of SIDS.

Stratum	Gene	SNP	Genotype Distribution	Additive (Y vs. X Alleles)		Dominant (YY/XY vs. XX Genotypes)		Recessive (YY vs. XX/XY Genotypes)	
			Case No. in SIDS (XX:XY:YY)	OR (95% CI) *	*p* Value *	OR (95% CI)	*p* value	OR (95% CI)	*p* Value
Female	*BCHE*	rs4680608	49:24:10	**0.609 (0.416~0.893)**	**0.010 (C)**	1.540 (0.939~2.524)	0.086	**3.717 (1.548~8.929)**	**0.004 (CC)**
Female		rs1355538	23:31:28	**1.501 (1.075~2.096)**	**0.016 (A)**	1.306 (0.767~2.222)	0.325	**2.309 (1.355~3.937)**	**0.002 (AA)**
Female		rs3495	36:32:15	**1.610 (1.140~2.272)**	**0.006 (C)**	**1.636 (1.008~2.656)**	**0.045 (CC/TC)**	**2.538 (1.282~5.025)**	**0.006 (CC)**
Female		rs1803274	46:33:04	0.683 (0.460~1.015)	0.058	**1.736 (1.064~2.834)**	**0.026 (TT/CT)**	1.091 (0.352~3.378)	1.000
Female		rs2048493	28:35:20	**1.562 (1.118~2.178)**	**0.008 (G)**	1.625 (0.982~2.686)	0.057	**2.179 (1.2~3.953)**	**0.009 (GG)**
Age 2–4 months		rs3495	39:24:12	0.774 (0.537~1.115)	0.168	1.157 (0.701~1.910)	0.569	**2.193 (1.055~4.566)**	**0.032 (CC)**
Age 2–4 months		rs4263329	62:10:03	0.757 (0.428~1.338)	0.337	1.124 (0.577~2.187)	0.732	**6.993 (1.145~41.667)**	**0.015 (GG)**
Autumn		rs2048493	15:17:11	**1.630 (1.049~2.564)**	**0.041 (G)**	1.544 (0.806~2.906)	0.254	**2.358 (1.065~4.950)**	**0.034 (GG)**
Autumn		rs4680608	29:09:05	1.248 (0.737~2.105)	0.465	1.071 (0.544~2.093)	0.862	**3.575 (1.328~10.820)**	**0.032 (CC)**
Autumn		rs12487357	35:06:02	1.486 (0.730~3.048)	0.303	1.202 (0.558~2.710)	0.662	**16.441 (1.852~238.6)**	**0.035 (CC)**
Summer	*ACHE*	rs10953305	09:18:03	1.482 (0.854~2.500)	0.178	1.159 (0.495~2.544)	0.676	**0.310 (0.097~0.984)**	**0.049 (AA)**

Note: Nominal *p* value < 0.05 and the respective effect sizes are marked in bold. OR * and *p* values * in the additive model were calculated using the linear-by-linear association of the Chi-square test. Alleles X and Y represent major and minor alleles, respectively. All nominal statistical significance did not retain significance after Bonferroni correction for multiple comparisons (adjusted α = 0.0001).

**Table 4:** Haplotypes of the *ACHE* gene with SIDS.

Haplotypes *		Frequencies in Controls ^#^	Frequencies in Overall SIDS	*p* Value	Frequencies in Male SIDS	*p* Value	Frequencies in Female SIDS	*p* Value
rs17880573	rs10953305							
C	G	49.9% (337:339)	48.7% (196:206)	0.707	47.8% (113:123)	0.586	50% (83:83)	0.987
C	A	34.9% (236:440)	36.6% (147:255)	0.581	37.8% (89:147)	0.438	35% (58:108)	0.994
T	A	14.8% (100:576)	14.6% (59:343)	0.935	14.3% (34:202)	0.870	14.9% (25:141)	0.953

Note: *p* value less than 0.05 was regarded as statistically significant. * Only haplotypes with frequency ≥ 0.01 were shown. ^#^ All the controls were included (females and males).

**Table 5:** Haplotypes of the *BCHE* gene with SIDS.

Haplotypes *					Frequencies in Controls ^#^	Frequencies in SIDS	*p* Value	Frequencies in Male SIDS	*p* Value	Frequencies in Female SIDS	*p* Value
rs3495	rs1803274	rs1355538	rs2048493	rs1126680							
T	C	G	C	C	57.3% (388:288)	56.1% (226:176)	0.707	63.1% (149:87)	0.121	46.3% (77:89)	**0.010**
C	T	A	G	C	10% (67:609)	11.9% (48:354)	0.316	9.7% (23:213)	0.904	15.1% (25:141)	0.058
C	C	A	G	C	8% (54:622)	8.9% (36:366)	0.598	8% (19:217)	0.920	12.5% (21: 145)	0.065
T	C	A	C	C	7.8% (56:620)	8% (32:370)	0.935	6.4% (15:221)	0.338	7.9% (13:152)	0.847
T	C	A	G	C	8.3% (53:623)	7% (28:374)	0.432	6.3% (15:221)	0.411	7.9% (13:153)	0.974
C	T	A	G	T	7.1% (48:628)	6.7% (27:375)	0.811	5.1% (12:224)	0.282	9% (15:151)	0.395

Note: *p* value less than 0.05 was regarded as statistically significant. * Only haplotypes with frequency ≥ 0.01 were shown. ^#^ All the controls were included (females and males).

## Data Availability

All raw data used in the study are available from the corresponding authors upon reasonable request.

## Supplementary Materials

The following supporting information can be downloaded at: https://www.mdpi.com/article/10.3390/genes15121656/s1, Table S1: SNPs with relevant alleles and genes as well as primer and probe sequences. Table S2: Quantitative Trait Loci (eQTL) Analysis on the selected SNPs in this study.
